# Biocide Potentiation Using Cinnamic Phytochemicals and Derivatives

**DOI:** 10.3390/molecules24213918

**Published:** 2019-10-30

**Authors:** Joana F. Malheiro, Jean-Yves Maillard, Fernanda Borges, Manuel Simões

**Affiliations:** 1 LEPABE, Laboratory for Process Engineering, Environment, Biotechnology and Energy, Faculty of Engineering, University of Porto, Rua Dr. Roberto Frias, 4200-465 Porto, Portugal; up201409754@fe.up.pt; 2 Cardiff School of Pharmacy and Pharmaceutical Sciences, Cardiff University, Cardiff, Wales CF10 3NB, UK; MaillardJ@cardiff.ac.uk; 3 CIQUP, Department of Chemistry and Biochemistry, Faculty of Sciences University of Porto, Rua do Campo Alegre, 4169-007 Porto, Portugal; fborges@fc.up.pt

**Keywords:** biocide, disinfection, phytochemicals, potentiation, sessile cells

## Abstract

Surface disinfection is of utmost importance in the prevention of bacterial infections. This study aims to assess the ability of ten phytochemicals and related derivatives as potentiators of two commonly used biocides—cetyltrimethylammonium bromide (CTAB) and lactic acid (LA). LA in combination with cinnamic, hydrocinnamic, α-methylcinnamic, and α-fluorocinnamic acids had a factional inhibitory concentration index (FICI) ≤ 1 for *Escherichia coli* and *Staphylococcus aureus*. Several phytochemicals/derivatives in combination with biocides improved the biocidal efficacy against early sessile bacteria. The most effective combination was LA with allyl cinnamate (2.98 ± 0.76 log CFU·cm^−2^ reduction) against *E. coli*. The combination with CTAB was successful for most phytochemicals/derivatives with a maximum bactericidal efficacy against sessile *E. coli* when combined with allyl cinnamate (2.20 ± 0.07 log CFU·cm^−2^ reduction) and for *S. aureus* when combined with α-methylcinnamic acid (1.68 ± 0.30 log CFU·cm^−2^ reduction). This study highlights the potential of phytochemicals and their derivatives to be used in biocide formulations.

## 1. Introduction

Clinical, veterinary, and food-related settings rely extensively on biocides and other antimicrobials to control microbial burden and provide hygienically safe environments, infrastructures, and products [1]. Infections caused by resistant bacteria are very difficult to treat and lead to an increase in hospitalization days and costs [2]. The European Union estimates that around 33,000 deaths per year are caused by antibiotic-resistant bacteria in which 75% are due to healthcare-associated infections (HAIs). In addition, healthcare costs and losses in productivity are estimated to be 1.5 billion per year [3,4]. A report published in 2009 estimated 25,000 deaths per year with an increase in 2.5 million extra days in hospital care representing a cost higher than EUR 900 million [5]. In the USA alone, 3.6 million cases of foodborne diseases and 36.5 million domestically acquired illnesses are estimated each year [6]. The most effective way to prevent HAIs and foodborne diseases is accomplished by regular cleaning and disinfection of surfaces as it prevents pathogens to firmly attach [7,8].

Biocides are extensively used as disinfectants in healthcare, animal farms, and food industry facilities, in household cleaning products, and as preservatives in several foods, pharmaceuticals or cosmetic industries [9,10,11,12]. However, the use of biocides for everyday purposes and the misuse of biocides in different industries have led to a continuous exposure of bacteria to sub-lethal concentrations, which results in sustained selective pressure and consequent development of resistance [9,13,14,15].

Organic acids and quaternary ammonium compounds (QACs) are among the different classes of biocides that are widely used [16,17,18,19,20,21]. Organic acids are a class of disinfectants particularly used in the food industry [9,22,23,24]. Particularly, lactic acid (LA) is a naturally occurring weak organic acid that is considered environmentally friendly and recognized as safe [17,22]. LA is used both as a spray to decontaminate areas and as an acidulant for low pH fruit juices and foods, although its liquid form is less active in comparison to the aerosolized one [25]. The effectiveness of LA as an antimicrobial is based on its ability to cross the plasma membrane as undissociated acid, to disrupt the membrane, inhibit metabolic reactions, disturb intracellular pH homeostasis, and promote the accumulation of toxic anions [22,26,27].

QACs are one example of biocides used in different areas, due to their hard-surface cleaning, deodorization and antimicrobial properties [25,28,29,30]. Cetyltrimethylammonium bromide (CTAB) is a QAC which mode of action is known to result in the rupture of the cell membrane, following an interaction with cell membrane lipids [29,31,32].

The antimicrobial properties of plant secondary metabolites (phytochemicals) have been used for centuries based essentially on ethnopharmacological knowledge. However, in the last decade, the interest in phytochemicals has re-emerged as a vast source of antimicrobial compounds [33,34,35,36,37,38,39]. The interest in phytochemicals is mainly due to them being environmentally friendly and having low toxicity to humans, but they are also harmful against pathogenic organisms at antimicrobial active concentrations [8,35,40,41,42]. In addition, their ability to increase permeability of bacterial membranes and kill a wide range of microorganisms has been reported [43,44,45].

The long term sub-lethal exposure to antimicrobials can lead to a selective pressure that potentiates the emergence and spread of bacterial strains with reduced susceptibility to commonly used biocides. This selective pressure can also result in cross-resistance, where resistant bacteria can also be resistant to certain clinically relevant antibiotics [1,46]. Studies on antimicrobial resistance have shown cases of bacteria that survived after exposure to presumably lethal concentrations of biocides [28,47]. The development of biocidal formulations, where two or more antimicrobials with different modes of action are mixed, is an interesting approach to potentiate bactericidal efficacy while negating emerging bacterial resistance. Biocidal formulations can also improve antimicrobial efficacy and, therefore, the probability of bacteria to survive disinfection exposure is diminished [40,46,48]. New antimicrobials and formulations must follow critical characteristics such as: (i) to be active at low concentrations; (ii) to have broad-spectrum; (iii) to be low cost; (iv) to have no impact on product usage and organoleptic properties; (v) to be surface compatible; vi) to have low or no toxicity; and vii) to be environmentally friendly [11].

The purpose of this study was to assess the ability of phytochemicals and their derivatives to potentiate the action of commonly used biocides. A selection of ten phytochemicals/derivatives was chosen, taking into consideration the data obtained by Malheiro et al. [49]—where their antimicrobial and quorum sensing inhibition properties were studied. Firstly, the phytochemicals/derivatives were tested in combination with CTAB and LA using the checkerboard method [50]. The efficacy of these combinations was also tested against 2 h adhered bacteria in an early sessile state. Moreover, the surface hydrophobicity was quantified to understand the interaction between the phytochemicals, their derivatives, and biocides with bacterial membranes.

## 2. Results

### 2.1. Phytochemicals/Derivatives Potentiate Biocides in Growth Control

The checkerboard method was used to test a wide range of concentrations of phytochemicals/derivatives and biocides (LA and CTAB). Among the concentrations tested, a combination of each phytochemical/derivative with each biocide was selected (Table 1). Cinnamyl alcohol and cinnamamide were the only phytochemicals/derivatives that did not potentiate any biocide (factional inhibitory concentration index (FICI) > 1). Cinnamic acid, allyl cinnamate, and all the phytochemicals/derivatives tested that have a modified side-chain (hydrocinnamic, α-methylhydrocinnamic, α-methylcinnamic, and α-fluorocinnamic acids) were able to potentiate LA (FICI ≤ 1). The combination of LA with cinnamic acid, hydrocinnamic acid or α-methylcinnamic acid had FICI ≤ 1 for *Escherichia coli* and *Staphylococcus aureus*, while α-fluorocinnamic acid was able to potentiate LA effect towards *E. coli* (FICI = 0.8), *S. aureus* (FICI = 0.7), and *E. hirae* (FICI = 1). Cinnamaldehyde and methyl trans-cinnamate were able to decrease the CTAB concentration needed to inhibit bacterial growth.

### 2.2. Biocide-Phytochemical/Derivative Combinations Reduced Early Sessile Bacteria

**Figure 1:**
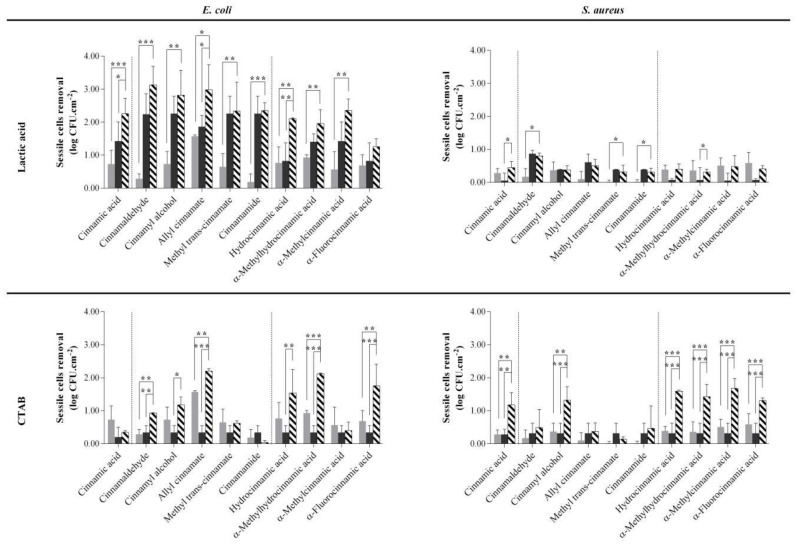
Effect of the combination of lactic acid (top) or CTAB (bottom) with the phytochemicals/derivatives on *E. coli* (left) and *S. aureus* (right). Each bacterium was exposed for 30 min to the established concentration of the phytochemical or derivative (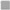) and biocide (lactic acid or CTAB; 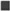) alone and in combination (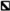). Bacteria were exposed to the phytochemicals/derivatives concentrations presented in Table 1. Values are mean ± SD. The statistical significance is represented (* *p* < 0.05; ** *p* < 0.01; *** *p* < 0.001).

Some of the combinations tested shown to potentiate bactericidal efficacy against the selected bacteria. Further studies were performed to understand the effects of such combinations against bacteria in an early sessile state. Two hours adhered bacteria were exposed to the phytochemical/derivative and the biocide, alone or in combination, for 30 min. When the phytochemicals/derivatives and LA were used alone, it was observed that *E. coli* CFU reduction was higher than for *S. aureus* (Figure 1). LA was particularly efficient in reducing the viability of sessile *E. coli.* The highest reduction was 2.26 log CFU·cm^−2^ after exposure to 40 mM LA. LA was only able to reduce *S. aureus* viability by 0.39 log CFU·cm^−2^ at a concentration higher than 40 mM, with a maximum reduction of 0.87 log CFU·cm^−2^ observed after exposure to 300 mM of LA. CTAB caused similar CFU reduction of *E. coli* and *S. aureus*. The efficiency of the majority of the combinations of phytochemicals/derivatives with LA on sessile *E. coli* (Figure 1, top left) and *S. aureus* (Figure 1, top right) was not significant (*p* > 0.05) in comparison with the exposure to LA. However, the CFU reduction of adhered *E. coli* when LA was used in combination with cinnamic acid was significant in comparison to the biocide (*p* < 0.05) and the phytochemical alone (*p* < 0.001). The same effect was observed when LA was combined with allyl cinnamate (*p* < 0.05) or hydrocinnamic acid (*p* < 0.01). On the other hand, CFU reduction efficiency of CTAB combined with phytochemicals/derivatives highlights some promising results (Figure 1 - below left for *E. coli* and below right for *S. aureus*). In fact, the combination of α-methylhydrocinnamic acid (*p* < 0.001) and α-fluorocinnamic acid (*p* < 0.001) with CTAB had higher efficiency in reducing CFU of sessile *E. coli* and *S. aureus* than the phytochemical/derivative or the biocide alone. The combination of cinnamaldehyde (*p* < 0.01) and allyl cinnamate (*p* < 0.01) with CTAB when used against sessile *E. coli* was more efficient than exposing the bacterium to these compounds or the biocide alone. For *S. aureus*, the combinations with increased efficiency were CTAB with cinnamic acid (*p* < 0.01), cinnamyl alcohol (*p* < 0.01), hydrocinnamic acid (*p* < 0.001), and methylcinnamic acid (*p* < 0.001). Considering the significant CFU reduction caused by the combination of a phytochemical/derivative with CTAB, the most significant effects were achieved using allyl cinnamate and α-methylhydrocinnamic acid—a sessile *E. coli* reduction of 2.20 ± 0.07 log CFU·cm^−2^ and 2.12 ± 0.03 log CFU·cm^−2^ were achieved, respectively. Exposing *S. aureus* to the combinations caused reductions of 1.68 ± 0.30 log CFU·cm^−2^, 1.59 ± 0.04 log CFU·cm^−2^, and 1.43 ± 0.37 log CFU·cm^−2^ when exposed to α-methylcinnamic acid (*p* < 0.001), hydrocinnamic acid (*p* < 0.001), and α-methylhydrocinnamic acid (*p* < 0.001), respectively.

### 2.3. Phytochemicals/Derivatives Effects on Bacterial Surface Hydrophobicity

The phytochemicals and derivatives causing the highest biocidal potentiation were selected to study their effects on the cell surface physico-chemical parameters, particularly the hydrophobicity. Allyl cinnamate was excluded as its antimicrobial activity was limited to *E. coli* and α-fluorocinnamic acid was not considered due to its high price (Table 2). Exposing *S. aureus* and *E. hirae* to the phytochemicals/derivatives for 30 min did not exert any significant alteration to the bacteria hydrophobicity or the surface tension parameters (*p* > 0.05) (Table 3). However, when studying their effects on *E. coli*, α-methylcinnamic acid was able to decrease the surface hydrophobicity (*p* < 0.01) after 30 min exposure. In addition, it decreased the apolar properties (*p* < 0.001) and increased the polar ones (*p* < 0.001) as well as the capacity to accept electrons (*p* < 0.001). α-Methylhydrocinnamic acid was also able to increase the polar properties (*p* < 0.001) and the ability to accept electrons (*p* < 0.05) by *E. coli* surface. Cinnamic acid increased *E. coli* polar properties and the capacity to accept electrons (*p* > 0.05).

## 3. Discussion

The use of biocides is essential to control the spread of pathogens in public and industrial settings. Biocide efficacy is affected by several factors, such as concentration, contact time, and environmental conditions under which it is applied [8,9,51,52]. Bacterial state, whether in suspension, adhered on a surface or in a biofilm, will impact on biocide efficacy [9,53]. The use of a biocide is a balance between a concentration that is low enough not to be hazardous during use or for the environment, and high enough to kill or inhibit bacteria and increasingly prevent the development of antimicrobial resistance [54].

A selection of ten phytochemicals/derivatives, previously evaluated for their antimicrobial and anti-quorum sensing, were selected for this study [33,49]. Taking into account the characteristics of the selected phytochemicals/derivatives (Table 2), this work was developed with the purpose of understanding their action in combination with biocides. Initially, LA and CTAB were tested in combination with each phytochemical/derivative and the concentration that induced growth inhibition was determined by the checkerboard method. Taking into consideration the concentration determined in this study for the biocides and phytochemicals/derivatives in combination, the effect of these combinations was assessed for the possibility to be used on CFU reduction of sessile bacteria. α-Methylhydrocinnamic acid and α-methylcinnamic acid modified *E. coli* surface properties. The effect of α-methylcinnamic acid was more evident when compared to α-methylhydrocinnamic acid with a decrease in bacteria hydrophilicity, which may be related to their structural properties, in particular, with molar refractivity. This property has been described as an indicator to optimize biological activity and is related to the real volume of the chemical and the London dispersion forces that influence chemical-biological interactions [55,56]. In this case, α-methylhydrocinnamic acid and α-methylcinnamic acid were the chemicals with the highest values of 46.54 cm^3^.mol^−1^ and 47.42 cm^3^.mol^−1^, respectively—hypothesizing the involvement of specific interactions with the membrane [57,58,59]. A higher susceptibility of *E. coli* compared to *S. aureus* when in contact with phytochemicals/derivatives was also observed by Malheiro et al. [33]. This result can be related to the presence of a thinner peptidoglycan layer in Gram-negative bacteria. In fact, phytochemicals/derivatives can be able to disturb and even disrupt the cell membrane structure. Moreover, they are able to cross the cell membrane by passive diffusion. In particular, those that are organic acids can increase bacterial membrane permeability, acidify the cytoplasm, and cause protein denaturation [60,61].

### 3.1. Phytochemicals/Derivatives Combination with Lactic Acid

The present data demonstrated that Gram-negative bacteria were generally more affected by the use of LA compared to the Gram-positive ones, corroborating previous studies [62,63]. The combination of LA with the phytochemicals/derivatives using the checkerboard method favored derivatives carrying a carboxylic group (cinnamic, hydrocinnamic, α-methylhydrocinnamic, and α-methylcinnamic acids). Although in this study a phosphate buffer of pH 7 was used, the use of LA may have affected the pH of the solution, lowering the pH of the combination to levels enough to modify the ratio of dissociate/undissociate forms of the organic acids—a parameter that can contribute to the destabilization of the cytoplasmic membrane [64,65]. In fact, pH is an important parameter when using organic acids like cinnamic, hydrocinnamic, α-methylhydrocinnamic, and α-methylcinnamic acids [60,61,66]. It is important to note, that lactic acid has a pKa of 3.79 and the mentioned organic acids a pKa around 4.34 [60,61,66]. This effect was observed with the combination of medium-chain fatty acids (caprylic, capric, and lauric acid) with organic acids (acetic, lactic, malic, and citric acids) against *Escherichia coli* O157:H7 [67]. LA may have caused physiological and morphological modifications in bacterial membranes, which may have facilitated the entrance of both LA and phytochemicals/derivatives into the cell [22]. The findings of Wang et al. [68] and Boomsma et al. [69] support this hypothesis, as they observed the efficacy of 0.5% LA on the inhibition of planktonic growth of *Salmonella* Enteritidis, *E. coli* and *Listeria monocytogenes.* Additionally, they also observed the release of intracellular proteins from these microorganisms following exposure to LA. In addition, LA antimicrobial action is known to be strongly dependent on the concentration of the acid and on the pH under which the experiment is carried out [70]. Some authors [16,25] demonstrated that under low pH, the biocide permeabilizes the outer membrane of Gram-negative bacteria. It was suggested that LA acts as a protonator of anionic components like the carbonyl and phosphate groups and consequently, the molecular interactions between components within the membrane are weakened [25,71].

LA combination with cinnamic acid (pKa 4.09) or hydrocinnamic acid (pKa 4.54) was able to reduce the CFU of sessile *E. coli*. In this case, the activity of these combinations may be related to the lower molecular weight of these two compounds among the phytochemicals/derivatives that have a carboxylic group (cinnamic acid 148.16 g/mol; hydrocinnamic acid 150.18 g/mol; α-methylhydrocinnamic acid 164.20 g/mol; α-methylcinnamic acid 162.19 g/mol; α-fluorocinnamic acid 166.15 g/mol) [49]. Allyl cinnamate effectiveness, when combined with LA, may be a result of its lipophilicity (logP of 3.17) and capacity to disturb membranes [68,72], as it has an additional Michael acceptor moiety in its structure, which can act as a covalent modifier affecting bacterial biosynthetic pathways and redox state [73,74].

### 3.2. Phytochemicals/Derivatives Combination with Cetyltrimethy Lammonium Bromide

Despite the lack of potentiation when using the checkerboard methodology, it was possible to observe growth inhibition when CTAB was used in combination with cinnamaldehyde and methyl trans-cinnamate, two phytochemicals/derivatives that have no carboxylic acid function. Membrane disruption and consequent leakage can be promoted by the CTAB mode of action, which may facilitate the access of the phytochemicals/derivatives and biocide to the cell cytoplasm, and consequent reaction with proteins and other cell components [75,76,77,78]. In fact, CTAB can bind to the negative cell surfaces of bacteria as a consequence of the electrostatic attraction by chemisorption, thus facilitating permeabilization [75,76,79]. Azeredo et al. [75] proposed that using a concentration of CTAB higher than its MBC, hydrophobicity and surface properties are increased and bacteria become hydrophilic and positively charged. After interacting with the membrane, CTAB promotes the disorganization of bacteria cell membrane and disruption [28,29]. The generation of reactive oxygen species during *E. coli* stress response to CTAB treatment has been previously reported [77]. In this study, both *E. coli* and *S. aureus* were affected by the combinations of phytochemicals/derivatives with CTAB. The mechanism of action of QACs is described as being primarily active against Gram-positive bacteria, but higher concentrations are also lethal to Gram-negative bacteria [25,80]. As described for the combination with LA, allyl cinnamate was able to potentiate CTAB action probably due to its lipophilicity (logP of 3.17) and capacity to disturb membranes facilitating CTAB access to *E. coli* cytoplasm [68,72,78]. The combination of cinnamaldehyde and CTAB was also able to potentiate the biocide against *E. coli*. Cinnamaldehyde is known to interact with the cell membrane components and also to enter the cell, modifying its components, such as enzymes and transcriptome, and promote cell death [81,82]. In addition, it can act as a reactive electrophile species and a substrate of the aldehyde dehydrogenase and disturb the bacteria detoxification pathways. According to Gill et al. [83], the action of cinnamaldehyde against *Listeria monocytogenes* and *E. coli* included a rapid inhibition of energy metabolism.

*S. aureus* was affected by the combinations with phytochemicals/derivatives that possess a carboxylic group giving them the ability to be hydrogen bond donors. This characteristic may be relevant for the interaction with the membrane and/or bacteria internal components together with the action of CTAB. The importance of the carboxyl and hydroxyl group for the activity of these chemicals was already described [33,49,60,84,85]. However, in this case, the importance of the combination with CTAB is highlighted since a similar effect was not observed when they were combined with LA.

Taking into consideration the results obtained by the combination with both LA and CTAB as well as previous data obtained by the same authors [33,49] for the phytochemicals/derivatives individually, six phytochemicals/derivatives stand out, i.e., cinnamic acid, cinnamaldehyde, hydrocinnamic acid, α-methylhydrocinnamic acid, α-methylcinnamic acid, and α-fluorocinnamic acid. However, α-fluorocinnamic acid was excluded from future combinations and formulation studies due to its high price in comparison with the phytochemicals/derivatives tested (Table 2). In fact, one important aspect in the development of a disinfection formulation is the cost of each product in order to commercialize a cost-efficient biocide [86].

## 4. Materials and Methods

### 4.1. Chemicals

Cinnamic acid and methyl trans-cinnamate were purchased from Merck (VWR, Portugal). CTAB, cinnamyl alcohol, hydrocinnamic acid, α-methylhydrocinnamic acid, and α-methylcinnamic acid were purchased from Acros Organics (Portugal). LA, cinnamaldehyde, allyl cinnamate, and α-fluorocinnamic acid were purchased from Sigma (Portugal). Cinnamamide was purchased from Alfa Aesar (VWR; Portugal). The CAS number of each chemical is provided in Table 2.

### 4.2. Microorganisms, Culture Conditions, and Test Solutions

*Staphylococcus aureus* NCTC 10788, *Escherichia coli* NCTC 10418, and *Enterococcus hirae* NCTC 13,383 were selected for this study, taking into consideration the model bacteria recommended by BS EN1276:2009 [87]. Bacterial cultures and phytochemicals/derivatives and biocides solutions were prepared as described by Malheiro et al. [49]. Phytochemicals and derivatives solutions were prepared using dimethyl sulfoxide (DMSO, Sigma). LA and CTAB were prepared using sterile distilled water. Neutralization was done using the universal neutralizer (lecithin 3 g.L^−1^, polysorbate 80 30 g.L^−1^, thiosulphate 5 g.L^−1^, L-histidin 1 g.L^−1^, saponin 30 g.L^−1^ in 1% phosphate buffer 0.25 M pH 7.2) for 10 min [87].

### 4.3. Bacterial Susceptibility by the Checkerboard Methodology

The checkerboard assay was performed accordingly to Abreu et al. [50] and Chan et al. [88] with some modifications. Bacterial suspensions (10^7^ CFU.ml^−1^) were prepared in fresh Mueller Hinton broth (MHB) diluted in phosphate buffer (0.02 M, pH 7). The assay was performed in a 96-well plate that was filled using electronic pipettes and a pipette robot (VIAFLO ASSIST together with INTEGRA’s VIAFLO electronic pipettes) to increase reproducibility. Phytochemical solutions (2.5% *v.v*^−1^) were added to a 96-well plate followed by the bacterial suspension and biocide solutions (2.5% *v.v*^−1^) in a total of 200 µL. Phytochemicals were tested in a range of 0 to 25 mM along the *y*-axis (rows) while CTAB was tested in the range of 0 to 0.1 mM and LA from 0 to 300 mM along the *x*-axis (columns). Plates were incubated for 24 h at 30 °C under 150 rpm of orbital agitation. The optical density at 600 nm was read before and after incubation. The minimum inhibitory concentration (MIC) of each biocide was considered when growth inhibition was observed [89]. The fractional inhibitory concentration index (FICI) was calculated according to Equation (1):

(1) $$FICI=\frac{\left[P\right]}{{\left[P\right]}_{MIC}}+\frac{\left[B\right]}{{\left[B\right]}_{MIC}}$$

where [*P*]/[*B*] is the concentration of the phytochemical/derivative (P) or biocide (B) in the combination, and ${\left[P\right]}_{MIC}/{\left[B\right]}_{MIC}$ are the MIC of each phytochemical and derivative or biocide alone.

The FICI and MIC were determined for each individual 96-well plate and the final FICI was determined considering three independent results. The phytochemicals/derivatives with a FICI < 1 were considered to potentiate the biocide since it highlights the reduction of the concentration of the phytochemical or biocide needed to inhibit bacterial growth when in combination (in comparison with their MIC alone). The concentration of the phytochemical or derivative in combination with the biocide was selected taking into consideration the best results obtained for at least two of the tested bacterium and the solubility. Three independent experiments were performed for each combination and bacterium.

### 4.4. Efficacy Against Early Sessile Cells

The bactericidal efficacy of the combination against early sessile bacteria was performed as described by Malheiro et al. [49]. Briefly, a bacterial suspension with 10^8^ CFU.ml^−1^ was allowed to adhere in a 96-well polystyrene microplate at 25 °C for 2 h and 150 rpm. After this time, the wells were washed with phosphate buffer (0.02 M, pH 7) and the adhered cells were exposed to the chemicals for 30 min at 25 °C under 150 rpm in order to mimic surface room temperature, according to Malheiro et al. [49]. For the purpose of this study, bacteria were exposed to the phytochemicals/derivatives, LA and CTAB individually, and in combination (phytochemicals/derivatives in combination with LA or CTAB), in the concentrations determined by the checkerboard method (Table 1). The added volume of phytochemical or derivative and biocide in combination was 10 µL (2.5% *v*/*v* of each). Afterward, the chemicals were removed and 200 µL of neutralizer was added. The neutralization process occurred for 10 min. The wells were washed with phosphate buffer (0.02 M, pH 7) and after the scrapping colony forming units (CFU) were assessed in Mueller-Hinton Agar (MHA). Three independent experiments were performed for each condition tested.

### 4.5. Bacterial Surface Hydrophobicity

The physicochemical properties of the bacteria under study were evaluated by the sessile drop contact angle measurement on bacteria lawns as described by Malheiro et al. [33] and Simões et al. [90] by using an OCA 15 Plus (DataPhysics, Filderstadt, Germany) video-based optical measuring instrument. In this study, the experiment was performed in phosphate buffer (0.02 M, pH 7) and the bacteria were exposed to the phytochemicals/derivatives for 30 min at the concentration established by the checkerboard method (Table 1). To assess the hydrophobicity, the van Oss method was used [91,92,93] as previously described by Malheiro et al. [33].

### 4.6. Statistical Analysis

The statistical program GraphPad Prism version 6 was used to analyze the data. One-way analysis of variance (one-way ANOVA) followed by the post hoc Dunnett’s multiple comparison test was used for analysis. A confidence level of ≥ 95% (*p* < 0.05), ≥ 99% (*p* < 0.01) and ≥ 99.9% (*p* < 0.001) was used as statistical significance. The results are presented as the average and standard deviation (SD) of three independent experiments for each sample.

## 5. Conclusions

Surface disinfection is a frontline strategy to control bacterial contamination and spread. In this study, a combinatorial approach has been considered to improve disinfection efficacy, where different antimicrobials with different modes of action were combined and their effectiveness in combination excels the individual. Overall, the combination of LA or CTAB with phytochemicals/derivatives was successfully accomplished. The combinations of LA with the phytochemicals/derivatives that possess a carboxylic group were able to inhibit the growth of *E. coli* and *S. aureus*. Phytochemicals/derivatives combination with LA only increased efficacy against *E. coli* sessile cells, and only with LA combined with cinnamic acid, allyl cinnamate or hydrocinnamic acid. CTAB was particularly successful in reducing CFU of sessile bacteria when combined with allyl cinnamate with α-methylcinnamic acid.

## Figures and Tables

**Table 1:** Phytochemicals/derivatives concentration used in combination with lactic acid (LA) or cetyltrimethylammonium bromide (CTAB).

Phytochemical or Derivative (mM)		Combination with			
		LA (mM)		CTAB (mM)	
		Concentration (mM)	Bacterium (FICI)	Concentration (mM)	Bacterium (FICI)
Cinnamic Acid	5	**20**	*E. coli* (0.8) *S. aureus* (0.9)	0.01	-
Cinnamaldehyde	0.5	300	-	**0.015**	*S. aureus* (0.9)
Cinnamyl alcohol	5	40	-	0.015	-
Allyl cinnamate	5	**200**	*E. coli* (1)	0.015	-
Methyl trans-cinnamate	5	40	-	**0.015**	*E. coli* (0.8)
Cinnamamide	5	40	-	0.015	-
Hydrocinnamic acid	8	**15**	*E. coli* (0.9) *S. aureus* (0.9)	0.015	-
α-Methylhydrocinnamic acid	5	**30**	*S. aureus* (1)	0.015	-
α-Methylcinnamic acid	3	**20**	*E. coli* (0.8) *S. aureus* (0.7)	0.015	-
α-Fluorocinnamic acid	5	**15**	*E. coli* (0.8) *S. aureus* (0.7) *E. hirae* (1)	0.015	-

These concentrations were determined by the checkerboard methodology where factional inhibitory concentration index (FICI) was calculated for all the tested bacteria and the best value for at least two bacteria was chosen. If no potentiation was detected, the combination was chosen, taking into consideration the phytochemical and derivative concentration used with both biocides and where solubility was not compromised. Potentiation with a given biocide is highlighted in bold and the FICI value is presented in parentheses.

**Table 2:** Technical information of the phytochemicals/derivatives and biocides used in this study.

Phytochemical or Derivative		Brand	CAS Number	Price per 1 g (€) ^a^
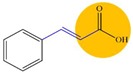				
	
	
Cinnamic Acid		Merck	140-10-3	4.86
Cinnamaldehyde	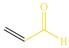	Sigma Aldrich	14371-10-9	0.05
Cinnamyl Alcohol	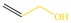	Acros Organics	104-54-1	0.15
Allyl Cinnamate	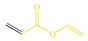	Sigma Aldrich	1866-31-5	0.49
Methyl Trans-Cinnamate	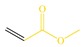	Merck	1754-62-7	0.12
Cinnamamide	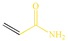	Alfa Aesar	621-79-4	7.22
Hydrocinnamic Acid	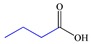	Acros Organics	501-52-0	0.31
α-Methylhydrocinnamic Acid	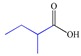	Acros Organics	1009-67-2	12.02
α-Methylcinnamic Acid	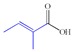	Acros Organics	1199-77-5	2.64
α-Fluorocinnamic Acid	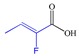	Sigma Aldrich	350-90-3	91.90
**Biocide**		**Brand**	**CAS number**	**Price per 1 g (€) ^a^**
CTAB		Acros Organics	57-09-0	0.26
Lactic Acid		Fluka	50-21-5	0.14

**^a^** The price per 1 g of product corresponds to the price of the chemicals when purchased by the research group. Some information was adapted from Malheiro et al. [49].

**Table 3:** Effect of the selected phytochemicals/derivatives on the surface tension parameters and hydrophobicity of *E. coli, S. aureus,* and *E. hirae*.

		Surface Tension Parameters (mJ m^−2^)												Hydrophobicity (mJ m^−2^)		
		$\gamma_{s}^{\mathrm{LW}}$			$\gamma_{s}^{\mathrm{AB}}$			$\gamma_{s}^{+}$			$\gamma_{s}^{-}$			$∆G_{\mathrm{sws}}^{\;}{}^{\mathrm{TOT}}$		
*E. coli*	Control (water)	33.43	±	1.98	13.74	±	3.65	1.03	±	0.52	48.78	±	3.08	28.98	±	4.49
	Control (DMSO)	31.99	±	1.36	15.78	±	1.99	1.26	±	0.32	50.11	±	4.16	29.91	±	4.98
	Cinnamic acid	29.67	±	3.33	21.63	±	1.76	2.45	±	0.34	47.81	±	1.49	24.65	±	1.64
	Cinnamaldehyde	32.96	±	0.61	12.78	±	2.02	0.92	±	0.27	45.02	±	3.13	24.84	±	3.73
	Hydrocinnamic acid	30.48	±	1.04	19.14	±	2.34	1.95	±	0.49	47.63	±	1.14	25.69	±	2.90
	α-Methylhydrocinnamic acid	28.55	±	1.19	**24.40**	**±**	1.86 ^**^	**3.00**	**±**	0.45 ^*^	49.87	±	0.68	25.75	±	0.80
	α-Methylcinnamic acid	**21.91**	**±**	4.42 ^***^	**31.60**	**±**	4.80 ^***^	**5.51**	**±**	1.76 ^***^	46.42	±	1.86	**19.08**	**±**	4.13 ^**^
*S. aureus*	Control (water)	35.26	±	1.18	18.01	±	2.09	1.71	±	0.52	48.68	±	4.23	25.80	±	5.62
	Control (DMSO)	36.24	±	1.19	17.56	±	0.68	1.58	±	0.09	48.92	±	4.14	25.80	±	4.73
	Cinnamic acid	34.79	±	1.73	19.35	±	0.75	1.83	±	0.14	51.07	±	0.66	27.94	±	1.31
	Cinnamaldehyde	35.70	±	0.52	17.25	±	3.06	1.51	±	0.51	50.19	±	2.08	27.76	±	3.06
	Hydrocinnamic acid	34.36	±	1.55	19.60	±	1.25	1.89	±	0.14	50.79	±	3.03	27.62	±	3.05
	α-Methylhydrocinnamic acid	34.49	±	2.20	16.93	±	0.90	1.34	±	0.09	53.59	±	2.47	32.40	±	3.09
	α-Methylcinnamic acid	35.82	±	1.04	16.68	±	1.09	1.32	±	0.18	52.98	±	1.21	31.34	±	2.18
*E. hirae*	Control (water)	35.65	±	1.77	13.20	±	2.62	0.86	±	0.33	52.45	±	2.79	32.88	±	3.80
	Control (DMSO)	33.93	±	0.59	17.72	±	2.40	1.52	±	0.52	53.02	±	3.10	31.58	±	4.83
	Cinnamic acid	32.26	±	2.25	20.96	±	1.62	2.15	±	0.34	51.38	±	1.27	28.31	±	1.34
	Cinnamaldehyde	33.02	±	1.72	20.03	±	3.58	2.00	±	0.75	51.64	±	2.64	28.94	±	4.11
	Hydrocinnamic acid	30.61	±	1.70	22.87	±	2.29	2.61	±	0.46	50.25	±	1.73	26.48	±	1.76
	α-Methylhydrocinnamic acid	33.15	±	0.50	18.76	±	2.37	1.69	±	0.50	53.41	±	3.69	31.70	±	5.47
	α-Methylcinnamic acid	34.33	±	2.72	17.22	±	2.81	1.41	±	0.40	53.17	±	2.43	31.75	±	3.02

Statistically significant values (* *p* < 0.05; ** *p* < 0.01;*** *p* < 0.001) when compared to the control of DMSO are highlighted in bold. Bacteria were exposed to the phytochemicals/derivatives concentrations presented in Table 1. Values are mean ± SD.
